# The central domain of UNC‐45 chaperone inhibits the myosin power stroke

**DOI:** 10.1002/2211-5463.12346

**Published:** 2017-12-10

**Authors:** Paul J. Bujalowski, Paul Nicholls, Eleno Garza, Andres F. Oberhauser

**Affiliations:** ^1^ Department of Biochemistry and Molecular Biology The University of Texas Medical Branch Galveston TX USA; ^2^ Baylor College of Medicine The University of Texas Medical Branch Galveston TX USA; ^3^ Department of Neuroscience and Cell Biology The University of Texas Medical Branch Galveston TX USA; ^4^ Sealy Center for Structural Biology and Molecular Biophysics The University of Texas Medical Branch Galveston TX USA

**Keywords:** chaperone, myosin, UNC‐45

## Abstract

The multidomain UNC‐45B chaperone is crucial for the proper folding and function of sarcomeric myosin. We recently found that UNC‐45B inhibits the translocation of actin by myosin. The main functions of the UCS and TPR domains are known but the role of the central domain remains obscure. Here, we show—using *in vitro* myosin motility and ATPase assays—that the central domain alone acts as an inhibitor of the myosin power stroke through a mechanism that allows ATP turnover. Hence, UNC‐45B is a unique chaperone in which the TPR domain recruits Hsp90; the UCS domain possesses chaperone‐like activities; and the central domain interacts with myosin and inhibits the actin translocation function of myosin. We hypothesize that the inhibitory function plays a critical role during the assembly of myofibrils under stress and during the sarcomere development process.

Force generation in striated muscle comes from the myosin motor domain, an 110‐kDa globular protein that allows conversion of the chemical potential energy in ATP into mechanical work. This complex protein is incapable of self‐folding and assembly. Instead, molecular chaperones work in a precise network to allow a nascent myosin polypeptide to be protected from aggregation and folded into its native and functional conformation. The UNC‐45B chaperone is associated with the proper folding and function of the sarcomeric myosin 1, 2, 3, 4.

UNC‐45B is built of three domains: the C‐terminal UCS domain, the central domain, and the N‐terminal TPR domain 2. The conserved UCS domain is alone capable to prevent aggregation of the chaperone client protein, myosin, and it is responsible for the chaperone‐like properties of UNC‐45B 5. Our binding studies indicated that the central domain also interacts with myosin but does not prevent myosin aggregation, and thus, it does not possess chaperone‐like activities 5. This different behavior and the fact that the central domain induces significant conformational changes within the myosin–UNC‐45B complex through allosteric interactions 5, suggest that the central domain performs a different function than the UCS domain.

The UNC‐45B protein has been identified as a factor that might play a critical role in several disease syndromes such as congenital heart diseases and cancers 6. Several studies have shown that UNC‐45B is required for myosin accumulation in sarcomeres of the myocardium and thus might be critical for human heart proper function 7, 8, 9, 10, 11.

We have previously shown that the UNC‐45B chaperone inhibits the actin translocation function of myosin, which is believed to play an important role during the assembly of myofibrils under stress or during the development process 12. The precise mechanisms by which UNC‐45B chaperones myosin and modulates myosin head function are unknown. Which are the key regions in UNC‐45B mediating its myosin‐chaperoning and actin gliding inhibiting functions? Based on the crystal structures and biochemical data on *Drosophila* 6, 13, 14, *Caenorhabditis elegans* 15, 16, 17 UNC‐45B and also based on the molecular dynamics and biophysical studies on human UNC‐45B 5, 18, 19, we hypothesize that the UCS domain independently interacts with the myosin head, prevents thermal aggregation, and maintains mechanically unfolded intermediates of the myosin head competent for refolding, underscoring its interaction with non‐native states. In contrast, the central domain is capable of binding to native myosin heads but unable to prevent misfolding 5. Hence, we further hypothesize that the chaperone function resides in the UCS domain and the blocking effect is largely mediated by the central domain. Here, we directly tested this hypothesis using the well‐established actin filament gliding assay, which is the minimal experimental system for studying actomyosin motility 20, 21, 22.

## Materials and methods

### Protein expression and purification

The full‐length *Mus musculus* UNC‐45B and its central and UCS domains were subcloned, expressed, and purified as described in a previous publication 5.

### Actin purification and labeling

Actin was purified from rabbit skeletal muscle 22, 23. Briefly, actin was extracted from actin acetone powder using 3 mm NaN_3_, 0.2 mm CaCl_2_, 0.2 mm ATP, and 0.5 mm DTT in 2 mm Tris, pH 8.5. In order to polymerize the actin, 50 mm KCl and 2 mm MgCl_2_ were added and then dialyzed against 50 mm KCl, 2 mm MgCl_2_, and 0.5 mm DTT in 10 mm Tris, pH 7.5, to maintain the polymerized state 22. The filaments were labeled using AlexaFluor 594 phalloidin (Life Technologies, Carlsbad, CA, USA) 12, 22.

### Myosin purification and subfragment‐1 preparation

Myosin was extracted from rabbit skeletal muscle and purified using the technique of Margossian and Lowey 24. Subfragment‐1 (or S1) was produced by chymotryptic digest of synthetic myosin filaments 25. The S1‐bearing supernatant was dialyzed against 50 mm imidazole pH 7.0, 0.3 mm EGTA, and 1 mm DTT and cleared by centrifugation. This supernatant was then adjusted to 150 mm NaCl and purified by size exclusion chromatography on a Sephacryl S‐300 column (GE Healthcare, Piscataway, NJ, USA). The purity and composition of full‐length myosin and the S1 subfragment proteins were confirmed by SDS/PAGE 26.

### Actin filament gliding assay

The actin gliding experiments were performed on nitrocellulose‐coated surfaces as previously described by our group 12. Briefly, nitrocellulose‐coated coverslips were prepared by depositing 1 μL of 1% nitrocellulose in amyl acetate on a glass cover slip and spreading evenly with a pipette tip. The nitrocellulose‐coated coverslip was then used to prepare a flow cell. Myosin S1 at 0.2 mg·mL^−1^ was applied in a TBS buffer for 2 min. This was then washed with buffer to remove the unbound fraction, and then, the surface was blocked with 1 mg·mL^−1^ BSA in G‐actin buffer for 3 min before being rinsed with wash buffer (20 mm MOPS, pH 7.4, 80 mm KCl, 5 mm MgCl_2_, and 0.1 mm EGTA).

The Alexa‐594‐phalloidin‐labeled actin was then added at a concentration of 20 nm in wash buffer and incubated for 1 min. The assay buffer consisting of wash buffer supplemented with 0.7% methylcellulose, 1 mm ATP, 0.1 mg·mL^−1^ glucose oxidase, 0.02 mg·mL^−1^ catalase, 2.5 mg·mL^−1^ d‐glucose, and 50 mm DTT was then washed to commence the experiment 22. The flow cell was imaged using a Nikon Eclipse TE2000 microscope (Nikon, Melville, NY, USA) with a Nikon 40X 1.3 NA objective and a CoolSnapHQ camera (Photometrics, Tucson, AZ, USA). Images were taken every 1–5 s, depending upon the speed of motion, with an exposure time of 200 ms per frame. For analysis, we used the Difference Tracker software (Babraham Bioinformatics, Cambridge, UK) in imagej (NIH, Bethesda, MD, USA). All of these experiments were performed at room temperature, which ranged from 19 to 23 °C.

### Myosin ATPase assay

ATP hydrolysis was measured using a colorimetric ATPase Assay Kit (Innova Biosciences, Cambridge, UK) following the manufacturer's instructions. Briefly, 0.2 μm myosin S1, 1 mm fresh ATP, and 0.4 mg·mL^−1^ actin filaments were mixed with 2 μm of UNC‐45B, central domain or UCS domain in 10 mm Tris, 50 mm KCl, 10 mm MgCl_2_, 0.2 mm CaCl_2_, and 1 mm DTT. Each condition was assayed using 100 μL aliquots of the reaction mix at each time point, promptly quenched, and then the absorbance was measured at 650 nm (using a Molecular Devices VersaMax Tunable Microplate reader, Molecular Devices, Silicon Valley, CA, USA). These reactions were carried out at 25 °C.

### Statistical analyses

Unless otherwise stated, data are reported as mean ± standard deviation. Student's paired *t*‐test (two‐tailed) was applied to determine the significance of the differences in ATPase activity in the absence or presence of the different UNC‐45B protein constructs, respectively. Statistical significance was assigned as not significant for *P >*  0.05 and *for *P* ≤ 0.05.

## Results

We utilized an actin gliding assay 12, 20, 21, 22 to test which domain of UNC‐45B is responsible for the blocking effect of UNC‐45B on myosin‐mediated actin translocation. We added different recombinant UNC‐45B protein constructs in the assay buffer of an actin gliding experiment with S1 myosin fixed to nitrocellulose‐coated coverslips. Using this assay, we measured an average actin filament gliding velocity of about 400 nm·s^−1^, a value that is in agreement with previous measurements 27.

**Figure 1:**
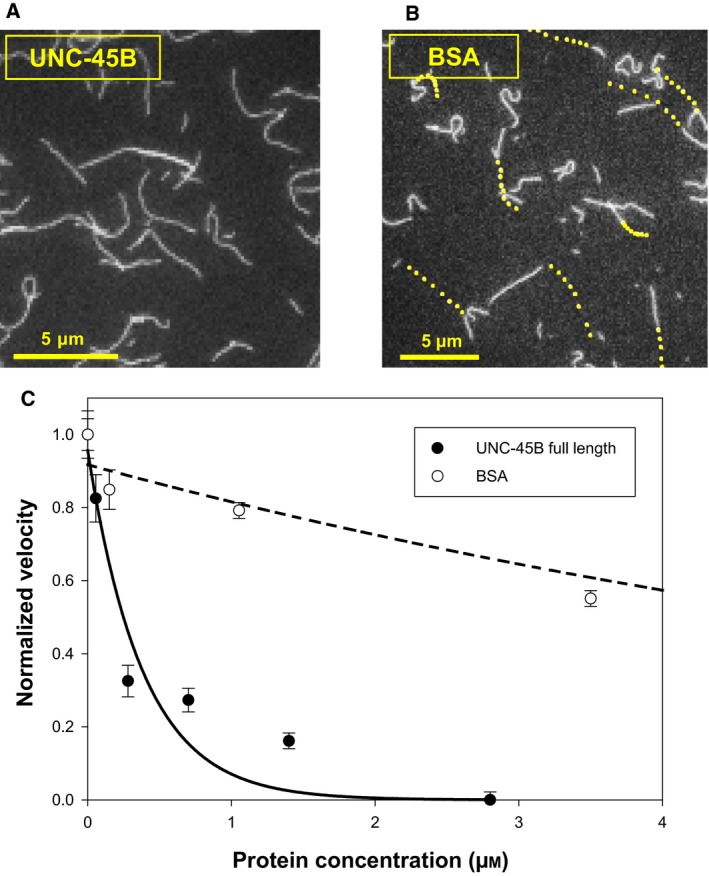
UNC‐45B specifically modulates myosin motor activity in an *in vitro* gliding assay. (A,B) Actin gliding assays with ATP‐bearing assay buffer supplemented with 3.5 μm BSA or 2.8 μm UNC‐45B, respectively. Each point represents the position of the trailing edge of an actin filament at 1‐s intervals. (C) Velocity of actin gliding in the presence of varying quantities of UNC‐45B and BSA. The solid lines correspond to a nonlinear least‐squares fit to single exponential decay equation (of the form *f* = *a* × exp(−*b* × *x*)) to the experimental data. All error bars represent standard deviation of three or more experiments.

Addition of 2.8 μm UNC‐45B completely inhibited the translocation of actin (Fig. 1A). As a control, we added BSA (3.5 μm) which showed a ~ 40% inhibition (Fig. 1B; each point represents the position of the trailing edge of an actin filament at 1‐s intervals). This nonspecific slowing effect of BSA in the gliding assay is most likely due to macromolecular crowding combined with the compromised power stroke of myosin S1, similar to the results obtained previously 12. Titrating UNC‐45B levels (Fig. 1C), we estimated the apparent *K*_d_ of this effect at about 0.2 μm.

**Figure 2:**
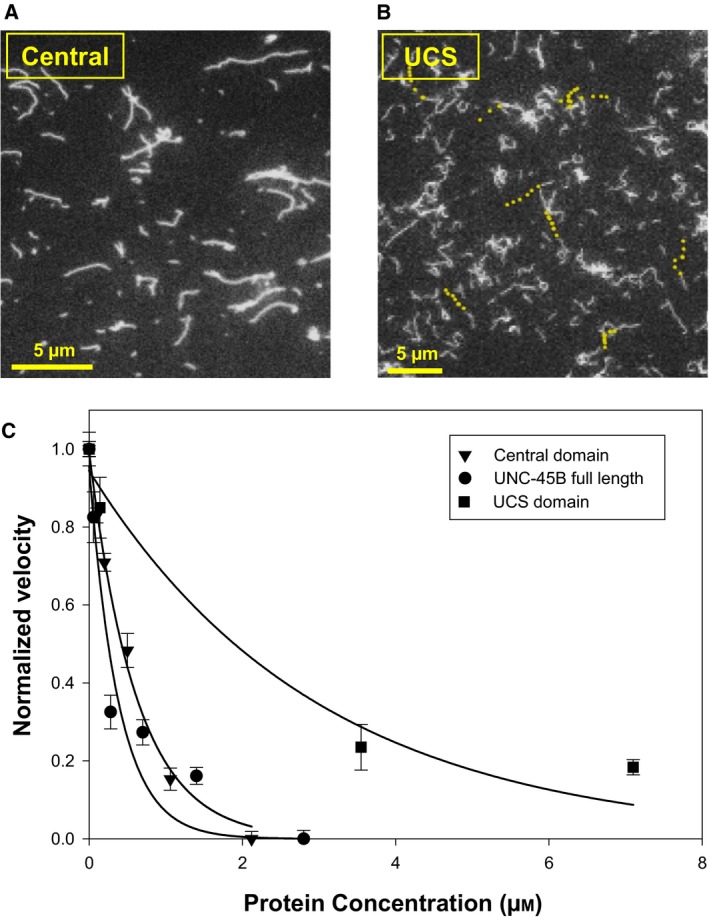
Addition of varying quantities of the central domain mimicked the inhibition curve of full‐length UNC‐45B, but the UCS domain did not. (A) 2.2 μm of central domain added to the assay buffer of a S1‐based gliding assay resulted in a 100% inhibition similar inhibitory effect to UNC‐45B. (B) 3.5 μm of the UCS domain added to the assay buffer resulted in a much weaker inhibition mimicking the BSA control. (C) Velocity of actin gliding in the presence of varying quantities of central domain (triangles), UCS domain (squares), and UNC‐45B (circles). The solid lines correspond to a nonlinear least‐squares fit to single exponential decay equation (of the form *f* = *a* × exp(−*b* × *x*)) to the experimental data. The UCS domain slowed actin translocation, although this effect plateaued. The central domain in contrast potently slowed the rate of actin translocation, similar to UNC‐45B full‐length controls. All error bars represent standard deviation of three or more experiments.

In our experiments, the addition of the central domain of UNC‐45B to the assay buffer resulted in a halt to actin gliding when added at a concentration of 2.2 μm (Fig. 2A). This result was similar to a positive control consisting of a similar concentration of full‐length UNC‐45B (Fig. 1A). The addition of 3.5 μm of the UCS domain resulted in a slowing of the actin gliding, although this effect was significantly weaker than the results observed with the central domain (Fig. 2B).

In order to estimate the apparent *K*_d_ of the inhibition by the central domain, we systematically increased its concentration in the gliding assay and estimated a value of 0.4 μm (Fig. 2C). Our results showed that the addition of the central domain was able to duplicate the effects of UNC‐45B. When we performed the same experiment with the UCS domain, the effect was much less potent and was more reminiscent of the effects of the BSA control (Fig. 1C). We hypothesize that the inhibitory effect of the UCS domain is nonspecific as it has higher affinity for myosin S1 than the central domain as measured by fluorescence binding assay 5. In that study, we found that estimated equilibrium dissociation constants for the UCS and central domain are 2.3 × 10^−7^ m and 1.6 × 10^−6^ m, respectively.

**Figure 3:**
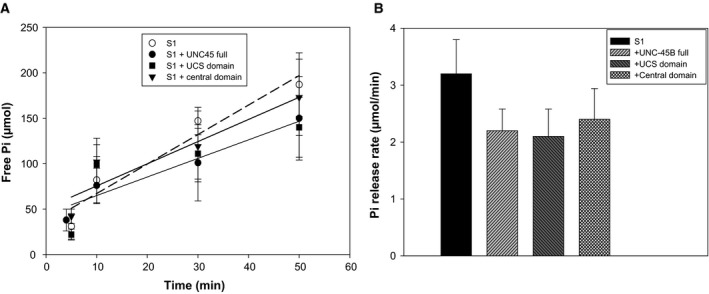
The actin translocation inhibitory property of the UNC‐45B central domain occurs through a mechanism that allows ATP turnover. (A) Kinetics of phosphate generation by myosin S1 (0.2 μm, open circles) mixed with 2 μm UNC‐45B (filled circles and no line), 2 μm UCS domain (filled squares), or 2 μm central domain (inverted triangles). The lines correspond to linear fits to the experimental data (S1 alone: dashed line; UCS domain: thin line; central domain: thick line). We found that a matching BSA concentration (2 μm) did not affect the S1 ATPase Pi release rate (data not shown). (B) Analysis of the rate of the myosin ATPase derived from linear fits to (A) showing that there is a small but not statistically significant difference (*P*‐value = 0.058) between the S1 ATPase Pi release rate with (2.4 ± 0.5 μmol·min^−1^) and without (3.2 ± 0.6 μmol·min^−1^) an inhibitory concentration of the central domain (2 μm). All error bars represent standard deviation of three or more experiments.

In order to test whether the central domain inhibits the myosin S1 actin‐activated ATPase, we measured the kinetics of phosphate generation by S1 (Fig. 3). We found that there is a small but not statistically significant difference between the kinetics of phosphate generation with (2.4 ± 0.5 μmol·min^−1^) and without (3.2 ± 0.6 μmol·min^−1^) an inhibitory concentration of the central domain (2 μm; Fig. 3B). Moreover, we found that full‐length UNC‐45B and the UCS domain do not inhibit the S1 actin‐activated ATPase (Fig. 3B). These data suggest that the actin translocation inhibitory property of the central domain of UNC‐45B occurs through a mechanism allowing ATP turnover while inhibiting the power stroke.

## Discussion and conclusions

**Figure 4:**
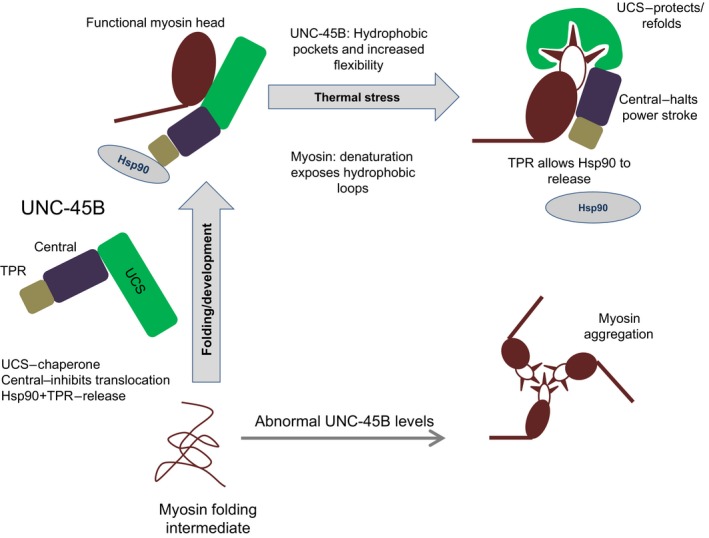
A novel model for the action of the UNC‐45B molecular chaperone in myosin assembly and repair. We propose that during myosin assembly, UNC‐45B assists the myosin head to attain its native conformation and subsequently locks it in a state that prevents premature power strokes; this translocation‐inhibited conformational state of myosin is relieved by Hsp90 upon successful assembly into the sarcomere. When a myosin head is damaged, Hsp90 molecules dissociate from the complex. Then, both the UNC‐45 chaperoning activity and blocking of the power stroke take place. Under these conditions, the aberrant behavior of the thick filament can then be corrected by refolding due to the chaperone network coordinated by their TPR domains. Hsp90 once again reassociates and the repaired thick filament may resume the organized generation of force.

Our previous studies showed that the myosin‐specific chaperone UNC‐45B inhibits the myosin motor domain power stroke 12. In this work, we have identified that the central domain acts alone as an inhibitor of myosin power stroke. Therefore, the UNC‐45B is a unique chaperone where each of the three domains has a clearly defined and different function (Fig. 4). The UCS domain binds to myosin heads and under stressful conditions stabilizes them and prevents their aggregation. The central domain has an inhibitory effect on the ability of myosin to translocate actin. We previously found that, on its own, Hsp90 had no effect on actin gliding, but when added after the UNC‐45B‐induced halt, it was capable of reversing it 12. Hence, the TPR domain binding to Hsp90 reverses the UNC‐45B inhibition of the myosin power stroke and resumes actin translocation.

What is the biological relevance of these observations? Based on these and our published data 5, 12, 19, 26, we propose a novel model for the action of the UNC‐45B molecular chaperone in myosin assembly and repair (Fig. 4). Considering the work by Gazda *et al*. 15 demonstrating that UNC‐45B forms a multimeric scaffold regulating the location of myosin heads and coordinating the activity of Hsp90, we believe that this UNC‐45B scaffold function is not merely limited to proper positioning of myosin head but it plays a multifunctional role during sarcomere formation and maintenance. We propose that an important function of UNC‐45B is to prevent the power stroke from occurring during myosin biogenesis. In the forming sarcomere, this would prevent untimely force from being applied to thin filaments, which could disrupt their orderly assembly into the semicrystalline sarcomere. Hsp90 could then serve both to assist in the completion of the myosin fold and to release the UNC‐45B‐mediated block, allowing the now fully formed myofibrils to contract normally.

According to this model, when myosin heads are damaged (e.g., due to thermal or chemical stress), Hsp90 molecules dissociate from the myosin/UNC‐45B/Hsp90 complex. Then, both the UNC‐45B chaperoning activity and blocking of the power stroke take place. This allows myosin refolding to occur without mechanical force displacing key components. Under this translocation‐inhibited conformational state of myosin, the aberrant behavior of the thick filament can then be corrected by refolding due to the chaperone network coordinated by their TPR domains. Hsp90 once again reassociates and the repaired thick filament may resume the organized generation of force. Hsp90 activity can be regulated by phosphorylation 28, which might switch on and off its modulation of UNC‐45B–myosin interactions for optimal control of myosin refolding and assembly 29.

According to this model, the UCS domain is the chaperone‐like domain, the central domain is an inhibitory domain that halts power stroke, and the TPR domain binds Hsp90 which releases UNC‐45B from thick filaments when myosin molecules obtain proper fold and are ready to return to performing its biological function.

The existence of a single chaperone molecule with different domains that have distinct functions on the client has not been reported previously, to our knowledge. Our studies should provide novel mechanistic insights into the pathogenesis of increased or reduced myosin activity (such as in certain hypertrophic and dilated cardiomyopathies) and its modulation by chaperones.

## Author contributions

PJB helped with the design and conduction of the experiments and wrote a draft of the manuscript. PN helped with the design and conducted experiments. EG conducted the ATPase experiments. AFO designed experiments, analyzed data and wrote the manuscript.

## References

[1] Barral JM , Bauer CC , Ortiz I and Epstein HF (1998) Unc‐45 mutations in *Caenorhabditis elegans* implicate a CRO1/She4p‐like domain in myosin assembly. J Cell Biol 143, 1215–1225. 9832550 10.1083/jcb.143.5.1215 PMC2133068

[2] Hutagalung AH , Landsverk ML , Price MG and Epstein HF (2002) The UCS family of myosin chaperones. J Cell Sci 115, 3983–3990. 12356904 10.1242/jcs.00107

[3] Barral JM , Hutagalung AH , Brinker A , Hartl FU and Epstein HF (2002) Role of the myosin assembly protein UNC‐45 as a molecular chaperone for myosin. Science 295, 669–671. 11809970 10.1126/science.1066648

[4] Hellerschmied D and Clausen T (2014) Myosin chaperones. Curr Opin Struct Biol 25, 9–15. 24440450 10.1016/j.sbi.2013.11.002 PMC4045384

[5] Bujalowski PJ , Nicholls P and Oberhauser AF (2014) UNC‐45B chaperone: the role of its domains in the interaction with the myosin motor domain. Biophys J 107, 654–661. 25099804 10.1016/j.bpj.2014.05.045 PMC4129474

[6] Lee CF , Melkani GC and Bernstein SI (2014) The UNC‐45 myosin chaperone: from worms to flies to vertebrates. Int Rev Cell Mol Biol 313, 103–144. 25376491 10.1016/B978-0-12-800177-6.00004-9 PMC4225561

[7] Epstein HF and Benian GM (2012) Paradigm shifts in cardiovascular research from *Caenorhabditis elegans* muscle. Trends Cardiovasc Med 22, 201–209. 23146617 10.1016/j.tcm.2012.07.021

[8] Benian GM and Epstein HF (2011) *Caenorhabditis elegans* muscle: a genetic and molecular model for protein interactions in the heart. Circ Res 109, 1082–1095. 21998299 10.1161/CIRCRESAHA.110.237685

[9] Wohlgemuth SL , Crawford BD and Pilgrim DB (2007) The myosin co‐chaperone UNC‐45 is required for skeletal and cardiac muscle function in zebrafish. Dev Biol 303, 483–492. 17189627 10.1016/j.ydbio.2006.11.027

[10] Geach TJ and Zimmerman LB (2010) Paralysis and delayed Z‐disc formation in the Xenopus tropicalis unc45b mutant dicky ticker. BMC Dev Biol 10, 75. 20637071 10.1186/1471-213X-10-75 PMC2919470

[11] Chen D , Li S , Singh R , Spinette S , Sedlmeier R and Epstein HF (2012) Dual function of the UNC‐45b chaperone with myosin and GATA4 in cardiac development. J Cell Sci 125, 3893–3903. 22553207 10.1242/jcs.106435 PMC3462083

[12] Nicholls P , Bujalowski PJ , Epstein HF , Boehning DF , Barral JM and Oberhauser AF (2014) Chaperone‐mediated reversible inhibition of the sarcomeric myosin power stroke. FEBS Lett 588, 3977–3981. 25240199 10.1016/j.febslet.2014.09.013

[13] Lee CF , Hauenstein AV , Fleming JK , Gasper WC , Engelke V , Sankaran B , Bernstein SI and Huxford T (2011) X‐ray crystal structure of the UCS domain‐containing UNC‐45 myosin chaperone from *Drosophila melanogaster* . Structure 19, 397–408. 21397190 10.1016/j.str.2011.01.002 PMC3060410

[14] Smith DA , Carland CR , Guo Y and Bernstein SI (2014) Getting folded: chaperone proteins in muscle development, maintenance and disease. Anat Rec (Hoboken) 297, 1637–1649. 25125177 10.1002/ar.22980 PMC4135391

[15] Gazda L , Pokrzywa W , Hellerschmied D , Lowe T , Forne I , Mueller‐Planitz F , Hoppe T and Clausen T (2013) The myosin chaperone UNC‐45 is organized in tandem modules to support myofilament formation in *C. elegans* . Cell 152, 183–195. 23332754 10.1016/j.cell.2012.12.025 PMC3549490

[16] Hoppe T , Cassata G , Barral JM , Springer W , Hutagalung AH , Epstein HF and Baumeister R (2004) Regulation of the myosin‐directed chaperone UNC‐45 by a novel E3/E4‐multiubiquitylation complex in *C. elegans* . Cell 118, 337–349. 15294159 10.1016/j.cell.2004.07.014

[17] Pokrzywa W and Hoppe T (2013) Chaperoning myosin assembly in muscle formation and aging. Worm 2, e25644. 24778937 10.4161/worm.25644 PMC3875649

[18] Fratev F , Osk Jonsdottir S and Pajeva I (2013) Structural insight into the UNC‐45‐myosin complex. Proteins 81, 1212–1221. 23408646 10.1002/prot.24270

[19] Bujalowski PJ , Nicholls P , Barral JM and Oberhauser AF (2015) Thermally‐induced structural changes in an armadillo repeat protein suggest a novel thermosensor mechanism in a molecular chaperone. FEBS Lett 589, 123–130. 25436418 10.1016/j.febslet.2014.11.034

[20] Kron SJ and Spudich JA (1986) Fluorescent actin filaments move on myosin fixed to a glass surface. Proc Natl Acad Sci USA 83, 6272–6276. 3462694 10.1073/pnas.83.17.6272 PMC386485

[21] Yanagida T , Nakase M , Nishiyama K and Oosawa F (1984) Direct observation of motion of single F‐actin filaments in the presence of myosin. Nature 307, 58–60. 6537825 10.1038/307058a0

[22] Sellers JR (2001) *In vitro* motility assay to study translocation of actin by myosin. Curr Protoc Cell Biol, Unit 13 2. 18228321 10.1002/0471143030.cb1302s00

[23] Spudich JA and Watt S (1971) The regulation of rabbit skeletal muscle contraction. I. Biochemical studies of the interaction of the tropomyosin‐troponin complex with actin and the proteolytic fragments of myosin. J Biol Chem 246, 4866–4871. 4254541

[24] Margossian SS and Lowey S (1982) Preparation of myosin and its subfragments from rabbit skeletal muscle. Methods Enzymol, 85(Pt B), 55–71. 6214692 10.1016/0076-6879(82)85009-x

[25] Weeds AG and Taylor RS (1975) Separation of subfragment‐1 isoenzymes from rabbit skeletal muscle myosin. Nature 257, 54–56. 125854 10.1038/257054a0

[26] Kaiser CM , Bujalowski PJ , Ma L , Anderson J , Epstein HF and Oberhauser AF (2012) Tracking UNC‐45 chaperone‐myosin interaction with a titin mechanical reporter. Biophys J 102, 2212–2219. 22824286 10.1016/j.bpj.2012.03.013 PMC3341559

[27] Toyoshima YY , Kron SJ , McNally EM , Niebling KR , Toyoshima C and Spudich JA (1987) Myosin subfragment‐1 is sufficient to move actin filaments *in vitro* . Nature 328, 536–539. 2956522 10.1038/328536a0

[28] Mollapour M , Tsutsumi S , Truman AW , Xu W , Vaughan CK , Beebe K , Konstantinova A , Vourganti S , Panaretou B , Piper PW *et al* (2011) Threonine 22 phosphorylation attenuates Hsp90 interaction with cochaperones and affects its chaperone activity. Mol Cell 41, 672–681. 21419342 10.1016/j.molcel.2011.02.011 PMC3062913

[29] Ni W , Hutagalung AH , Li S and Epstein HF (2011) The myosin‐binding UCS domain but not the Hsp90‐binding TPR domain of the UNC‐45 chaperone is essential for function in *Caenorhabditis elegans* . J Cell Sci 124, 3164–3173. 21914819 10.1242/jcs.087320 PMC3706032

